# Discovery of a potential bladder cancer inhibitor CHNQD-01281 by regulating EGFR and promoting infiltration of cytotoxic T cells

**DOI:** 10.1007/s42995-024-00246-w

**Published:** 2024-08-05

**Authors:** Jian-Yu Liu, Yao-Yao Jiang, Peng-Jie Li, Bo Yao, Yi-Jing Song, Ji-Xiu Gao, Gulab Said, Yang Gao, Jun-Yu Lai, Chang-Lun Shao

**Affiliations:** 1 Key Laboratory of Marine Drugs, the Ministry of Education of China, School of Medicine and Pharmacy, Ocean University of China, Qingdao, 266003 China; 2 Department of Critical Care Medicine, the Affiliated Hospital of Qingdao University, Qingdao, 266003 China; 3 Department of Chemistry, Women University Swabi, Swabi, 23430 Pakistan; 4 Laoshan Laboratory, Qingdao, 266237 China; 5 Key Laboratory of Tropical Medicinal Resource Chemistry of Ministry of Education, College of Chemistry and Chemical Engineering, Hainan Normal University, Haikou, 571158 China

**Keywords:** Bladder cancer, Marine natural products, Brefeldin A, EGFR/PI3K/AKT, EGFR/ERK, T-cell infiltration

## Abstract

As one of the common malignancies that threaten human life, bladder cancer occurs frequently with a high mortality rate in the world, due to its invasion, recurrence and drug resistance. Natural products from marine microorganisms are becoming the hotspots in discovery of new candidate drug entities, especially in the area of cancer. Brefeldin A (BFA) is a natural Arf-GEFs inhibitor, but due to the low aqueous solubility, strong toxicity, and poor bioavailability, it is urgent to conduct structural optimization research. Herein, a new BFA pyridine acrylate derivative **CHNQD-01281** with improved solubility was prepared and found to exert moderate to strong antiproliferative activity on a variety of human cancer cell lines. It was noteworthy that **CHNQD-01281** was most sensitive to two bladder cancer cell lines T24 and J82 (IC_50_ = 0.079 and 0.081 μmol/L) with high selectivity index (SI = 14.68 and 14.32), suggesting a superior safety to BFA. In vivo studies revealed that **CHNQD-01281** remarkably suppressed tumor growth in a T24 nude mice xenograft model (TGI = 52.63%) and prolonged the survival time (ILS = 68.16%) in an MB49 allogeneic mouse model via inducing infiltration of cytotoxic T cells. Further mechanism exploration indicated that **CHNQD-01281** regulated both EGFR/PI3K/AKT and EGFR/ERK pathways and mediated the chemotactic effect of chemokines on immune effector cells. Overall, **CHNQD-01281** may serve as a potential therapeutic agent for bladder cancer through multiple mechanisms.

**Supplementary Information:**

The online version contains supplementary material available at 10.1007/s42995-024-00246-w.

## Introduction

Globally, bladder cancer (BC) is one of the top ten malignant tumors, which affects three to four times as many men as women and is the fourth most common malignancy in men (Compérat et al. 2022; Lenis et al. 2020; Sung et al. 2021). BC is the most common genitourinary cancer in China, with a poor prognosis and high prevalence. The disease progresses rapidly, and metastasis occurs in the early phase with more than 90% of patients eventually succumbing to the consequences of local invasion and metastasis (Hu et al. 2023; Lee et al. 2023). At present, surgery and chemotherapy are the main treatment methods for BC, while neither of which has shown much benefit. Moreover, postoperative recurrence occurs in 70% of BC patients, and the recurrence rate is still around 40% after adjuvant chemotherapy (Hu et al. 2023). Both invasion and recurrence are the major reasons that limit the therapeutic prognosis of BC.

To improve the prognosis, reduce the recurrence and prolong the survival duration of patients with BC, immunotherapy and targeted therapies have become promising options for patients with various stages of disease, such as checkpoint inhibitors, adoptive cell therapy, cytokine-based therapy, and bispecific antibodies and antibody–drug conjugates (Dyrskjøt et al. 2023). Clinically, the administration of Bacillus Calmette Guerin (BCG) immunotherapy for patients with high-risk non-muscle-invasive bladder cancer has been the gold-standard adjuvant for prevention of recurrence and progression (Lenis et al. 2020; Pettenati et al. 2018). However, unsatisfactory effects, apparent resistance, and even severe side effects in different populations were ascribed to the heterogeneity in BCG immunotherapy.

Therefore, with the growing understanding of the pathogenesis of bladder cancer, new drug treatment is urgently needed. The contributions of marine natural products to the development of present drugs have been extensively documented (Chen et al. 2023; Guo et al. 2023; Hai et al. 2021, 2022; Zou et al. 2023). A considerable increase in the number of marine drugs approved for clinical use has emerged in the past few decades, as well as a large number of marine-derived molecules being investigated in preclinical and clinical studies (Newman et al. 2020). Brefeldin A (BFA), a naturally occurring 13-membered macrolactone with a cyclopentane substituent, was obtained from various fungi species as a metabolite. Studies show that BFA performs potent antiproliferative efficacy against many human cancer cell lines with IC_50_ values at nanomolar level and thus has been selected by the National Cancer Institute (NCI) for detailed preclinical survey, revealing that BFA dramatically suppresses tumor growth and prolongs the survival duration in several mouse models (Sausville et al. 1996). However, BFA has a short half-life, strong toxicity, and poor solubility. These disadvantages limit its further clinical application. Therefore, the development of BFA-related derivatives with improved aqueous solubility, high safety, and favorable pharmacokinetic (PK) properties are particularly important.

**Fig. 1:**
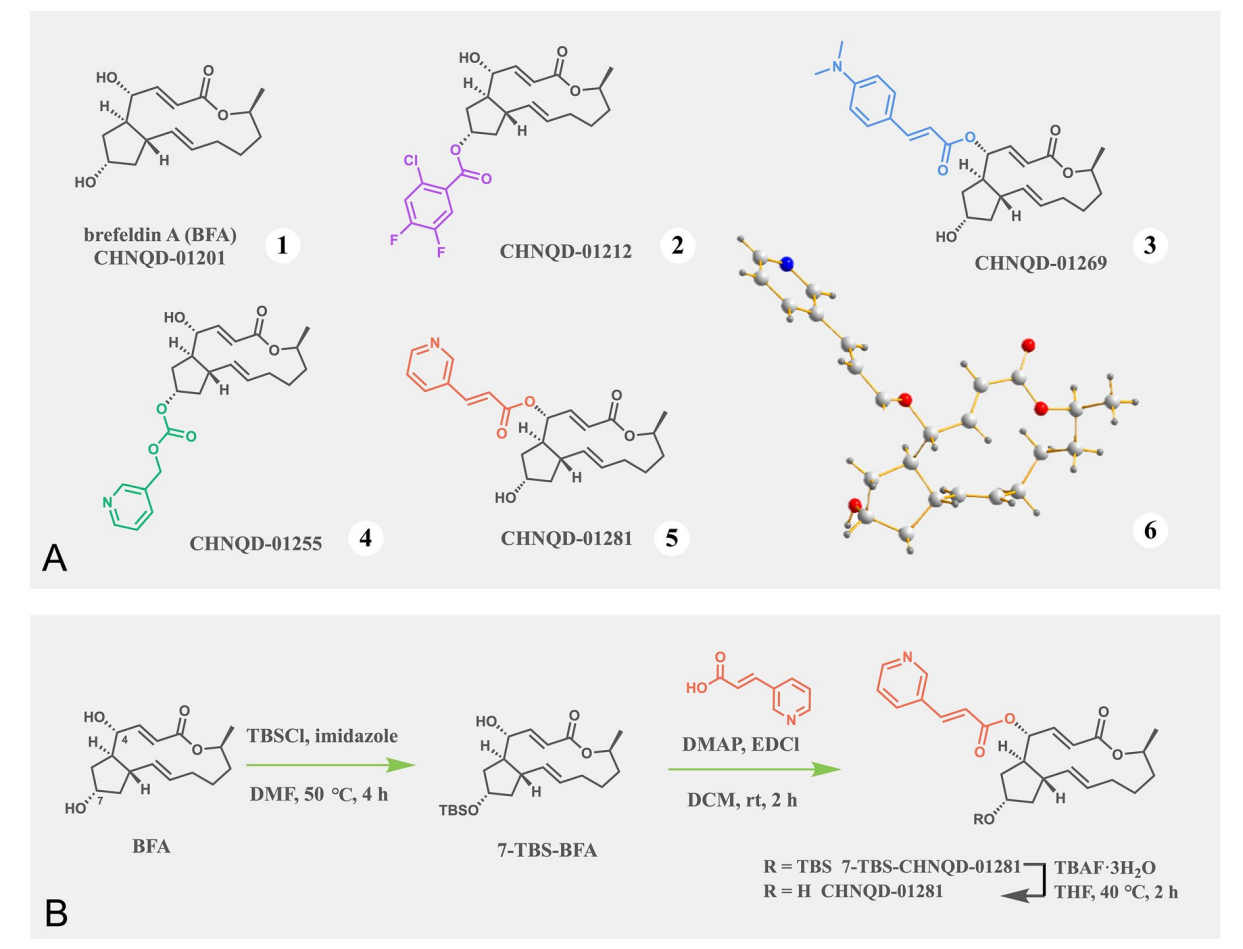
BFA and its derivatives. **A** BFA **(1)** and its derivatives **(2–4)** with antitumor effects published in our laboratory, the chemical **(5)** and X-ray crystallographic structure **(6)** of **CHNQD-01281**. **B** The synthetic route of **CHNQD-01281**

As part of our continuing program to discover bioactive marine natural products and potent drug leads from marine-derived microorganisms (Chao et al. 2021; Han et al. 2023; Hou et al. 2019; Jia et al. 2015; Shao et al. 2011; Xu et al. 2022), BFA (CHNQD-01201, Supplementary Figs. S1−S3) was obtained using an activity-directed strategy, combined with fingerprinting analysis, from the medicinal mangrove *Acanthus ilicifolius*-derived fungus *Penicillium* sp. (CGMCC No.17193) (Wang et al. 2022a). Subsequently, a series of BFA-derivatives were rationally designed and synthesized, constructing a small compound library in our group. Notably, some of BFA ester and carbonate derivatives, such as the benzoic acid ester CHNQD-01212 (Lu et al. 2022), the cinnamic acid ester CHNQD-01269 (Jiang et al. 2022a) and the carbonate CHNQD-01255 (Jiang et al. 2022b) (Fig. 1A), demonstrated superior antitumor activities to various cancers. These findings indicated that modification of the hydroxyl groups of BFA providing a promising method to offer antitumor agents with significant therapeutic effects or superior druggability properties.

In the current research, a new BFA pyridine acrylate derivative **CHNQD-01281** was synthesized, and its antiproliferative activity was evaluated on a variety of human cancer cell lines. It was found that **CHNQD-01281** was highly sensitive to bladder cancer cells. Subsequently, we comprehensively investigated the inhibitory effect and the underlying mechanisms of **CHNQD-01281** on bladder cancer in vitro and in vivo. Further studies including a solubility assay in aqueous solution, tissue distribution and safety profiling, were also conducted to validate its potential efficacy.

## Material and methods

### Chemistry

#### General experimental procedures

NMR spectra were recorded on a JEOL JEMECP NMR spectrometer (500 and 400 MHz for ^1^H NMR, 125 and 100 MHz for ^13^C NMR). ESIMS and HRESIMS spectra were obtained from a Micromass Q-TOF spectrometer (Waters Ltd.) and a Thermo Scientific LTQ Orbitrap XL spectrometer. X-ray crystal data were obtained on Agilent Gemini Ultra diffractometer (Cu Ka radiation). Semi-preparative HPLC analysis was performed on a Hitachi L-2000 system (Hitachi Ltd.) using a C18 column [(Eka Ltd.) Kromasil 250 mm × 10 mm, 5 μmol/L, 2.0 mL/min]. Silica gel (Qing Dao Hai Yang Chemical Group Co.; 200−300 mesh) was used for column chromatography.

#### Synthetic and characterization of CHNQD-01281

Natural BFA was isolated from the fermentation of the endophytic fungus *Penicillium* sp. (CGMCC No.17193) collected from the medicinal mangrove *Acanthus ilicifolius* in our previous work (Wang et al. 2022a). With sufficient BFA in hand, subsequent protection of 7-OH of BFA was completed with TBSCl to furnish 7-TBS-BFA (Supplementary Figs. S4−S5). Next, 3-(pyridin-3-yl)acrylic acid (37.8 mg, 0.25 mmol) was added to a solution of 7-TBS-BFA (100.0 mg, 0.25 mmol), DMAP (31.0 mg, 0.25 mmol) and EDCl (193.6 mg, 1.0 mmol) in 10 mL dry dichloromethane (DCM). The reaction mixture was stirred at 45 °C for 2 h, then the reaction mixture was quenched with water. The organic layer was concentrated and the residue containing 7-TBS-CHNQD-01281 without further purified was then dissolved in anhydrous THF and reacted with TBAF·3H_2_O (3 equivalent) at 40 °C for 2 h. The mixture was dried under vacuum to remove THF, then extracted by DCM/H_2_O, and the organic layer was purified by silica gel column chromatography followed by semi-preparative HPLC to yield the target.

Brefeldin A 4-*O*-3-(pyridin-3-yl) acrylate **(CHNQD-01281**): white powder, yield 22%. [*α*]^20^_D_ + 100.0 (*c* 0.1, MeOH); UV (MeOH) *λ*_max_ (log ε) 196 (2.28), 259 (2.02) nm; IR (KBr) *ν*_max_ 2932, 1711, 1641, 1258, 1169, 978 cm^–1^; ^1^H NMR (400 MHz, CDCl_3_) *δ* 8.74 (1H, s), 8.61 (1H, d, *J* = 4.9 Hz), 7.85 (1H, dt, *J* = 8.0, 2.0 Hz), 7.70 (1H, d, *J* = 16.1 Hz), 7.35 (1H, dd, *J* = 8.0, 4.8 Hz), 7.29 (1H, dd, *J* = 15.7, 3.3 Hz), 6.54 (1H, d, *J* = 16.1 Hz), 5.79–5.64 (2H, overlapped), 5.39 (1H, dt, *J* = 10.6, 2.6 Hz), 5.31 (1H, dd, *J* = 15.5, 9.8 Hz), 4.83 (1H, m), 4.33 (1H, m), 2.44 (1H, m), 2.32–2.17 (2H, overlapped), 2.01–1.96 (2H, overlapped), 1.90–1.80 (3H, overlapped), 1.79–1.61 (2H, overlapped), 1.59–1.46 (2H, overlapped), 1.23 (3H, d, *J* = 6.2 Hz), 0.93 (1H, m); ^13^C NMR (100 MHz, CDCl_3_) *δ* 166.3 (C = O), 166.0 (C = O), 151.5 (CH), 151.1 (CH), 149.8 (CH), 141.1 (CH), 136.0 (CH), 134.4 (CH), 131.2 (CH), 123.9 (C), 120.7 (CH × 2), 117.9 (CH), 75.9 (CH), 75.8 (CH), 71.9 (CH), 52.3 (CH), 44.1 (CH), 40.3 (CH_2_), 38.8 (CH_2_), 34.3 (CH_2_), 31.9 (CH_2_), 26.8 (CH_2_), 21.0 (CH_3_); HRESIMS *m/z* 412.2124 [M + H]^+^ (calcd. for C_24_H_30_NO_5_^+^, 412.2118).

#### X-ray crystallographic analysis of CHNQD-01281

Colorless crystals of the target was obtained from MeOH via slow evaporation. The crystal data was collected at 293 K on an Agilent Gemini Ultra diffractometer with Cu Ka radiation (λ = 1.54184 Å). Direct methods (SHELXS-97) and full-matrix least-squares difference Fourier techniques were used to solve and refine the structure. All non-hydrogen atoms were anisotropically refined. The data has been deposited at the Cambridge Crystallographic Data Centre with the deposition number 2321665, which can be obtained, free of charge, on application to the Director, CCDC, 12 Union Road, Cambridge CB21EZ, UK.

Crystal data for **CHNQD-01281:** C_24_H_29_NO_5_, Mr = 411.48, monoclinic, space group P 21 with a = 6.0555 (8) Å, b = 10.9148 (14) Å, c = 17.554 (2) Å, *α* = 90°, *β* = 98.718 (13)°, *γ* = 90°, V = 1146.8 (3) Å3, Z = 2, Dx = 1.192 g/cm^3^, *μ* (Cu K*α*) = 0.675 mm^−1^, and F (000) = 440. Crystal dimensions: 0.12 × 0.12 × 0.11 mm^3^. Independent reflections: 2848 (*R*int = 0.0461). The final *R*1 values were 0.0246, Flack parameter = -0.1 (34), *ω*R2 = 0.1185 (I > 2ζ (*I*)).

### Molecular reagents

#### Cell culture and reagents

Human tumor cell lines Eca-109, BEL-7402, BxPC-3, HCT-116, HT-29, T24, HCT-8, SGC-7901, DLD-1, TE-1, BT-549, MGC-803 and human hepatocyte cell line L-02 were cultured in Roswell Park Memorial Institute (RPMI) 1640. HuH-7 and SW-480 were cultured in Dulbecco’s modified Eagle’s medium (DMEM). J82, HepG2, Hela, U87 were cultured in Minimum Essential Medium (MEM). Murine bladder cancer cell line, MB49, was cultured in DMEM. The above-mentioned culture medium was provided by GIBCO (Grand Island, USA), contained 10% fetal bovine serum (PAN, Germany), 10 mg/mL of streptomycin, and 10 kU/mL penicillin (Beyotime, China).

#### Animals

Specific pathogen free (SPF)-grade C57BL/6 mice and BALB/c-nu mice were purchased from Jinan Pengyue Experimental Animal Center (license No. SCXK 20190003). The protocol was approved by the Research Review Committee of the Ocean University of China. All mice were housed in 12-h light and 12-h dark conditions in which the humidity was 50% ± 10% and the temperature was 22 ± 2 °C. All mice had free access to food and water. The human bladder cancer cell line T24 was injected subcutaneously into BALB/c-nu mice to construct a xenograft tumor model. The cell suspension of mouse bladder cancer cell line MB49 was injected into C57BL/6 mice subcutaneously to construct a syngeneic tumor model.

#### Cell proliferation assay

Cells were seeded in 96-well plates and incubated with indicated concentrations of **CHNQD-01281** for indicated periods. Next, medium was discarded and MTT (5 mg/mL) was added to each well. The cells were incubated at 37 °C for another 4 h. After that, supernatant was discarded, and DMSO was added and incubated at 37 °C for 15 min. Finally, absorbance was measured at 570 nm (Molecular Devices, USA).

#### Colony formation assay

T24 and J82 cell lines were seeded in 6-well plates at 500 cells/well. The cells were cultured overnight in medium. Compound **CHNQD-01281** was added according to the concentration gradient, and the cells were placed in a 37 °C cell incubator containing 5% CO_2_ for 12 days. The cells were then fixed with 4% paraformaldehyde for 15 min and stained with crystal violet for 30 min. After that, pictures were taken and colony numbers were counted. The clone formation rate relative to the solvent control group was calculated.

#### Cell cycle analysis

T24 and J82 cell lines were seeded in 6-well plates (3 × 10^5^ cells/well) and treated with different concentrations of **CHNQD-01281** or DMSO. Cells were then harvested, washed with ice-cold PBS, and fixed in 70% cold ethanol overnight at − 20 °C. Fixed cells were then collected, washed, and stained with 200 *μ*L of Muse™ cell cycle reagent for 30 min in the dark at room temperature. The cell cycle distribution was immediately analyzed by Muse Cell Analyzer (Millipore, Billerica, MA, USA).

#### Cell apoptosis analysis

T24 and J82 cell lines were seeded in 6-well plates (3 × 10^5^ cells/well) and treated with different concentrations of **CHNQD-01281** or DMSO. Muse™ Annexin V and Dead Cell assay kit (Muse TM Cell Analyzer, Millipore (catalog no. MCH100105)) was used to determine the cell apoptosis by Muse cell analyzer (Millipore, Billerica, MA, USA).

#### Western blot

T24 and J82 cell lines were lysed in protein lysis buffer, which included phosphatase inhibitors and a cocktail of protease inhibitors. A BCA kit (SparkJade, China) was used to determine the total protein concentration. After protein denaturation, SDS-PAGE electrophoresis was performed and the protein transferred from the gel to a PVDF membrane (Millipore, USA). Then primary antibodies against cleaved caspases-3, cleaved PARP, AKT, phosphorylated AKT, ERK1/2, phosphorylated ERK1/2, EGFR, mTOR, phosphorylated mTOR and Tubulin (CST, USA) were diluted at 1:1000 at 4 °C overnight. The membranes were then washed and incubated with secondary antibodies dissolved in 5% nonfat milk. A chemiluminescence imaging system was used to detect the signals (Tanon, China).

#### Immunohistochemistry (IHC) and immunofluorescence (IF)

Fixed tissues were embedded in paraffin and then sectioned. The specimens were incubated with antibodies against Ki67, CD3, CD4, CD8 (Servicebio, China) and EGFR (ABclonal, China) at 4 °C overnight. The next day, the slides were washed with 1 × PBST three times for 5 min each and stained with the secondary antibodies and Hoechst 33342 dye (Beyotime, China) for 60 min for IF. For IHC staining, the sections were developed with a rabbit/mouse HRP kits (DAB) (Beyotime, China) according to the manufacturer’s instructions.

#### RNA-seq analysis

The quality of RNA samples was assessed by agarose gel electrophoresis and using the Agilent 2100 Bioanalyzer (Agilent Technologies). Purified RNA was subjected to cDNA library construction and high throughput sequencing on an Illumina HiSeq Xten platform by Majorbio Biotech. SAM analysis was applied to calculate differential gene expression. Identified genes with significant upregulation and downregulation were mapped (fold change ≥ 2 and *P* < 0.05). Majorbio I-Sanger Cloud Platform (http://www.i-sanger.com) was used for data analysis.

#### Statistical analyses

Statistical analyses were performed using IBM SPSS Statistics 24 and GraphPad Prism 9.0 (San Diego, CA). Statistical comparisons were performed using one-way analysis of variance (ANOVA) followed by post hoc Tukey's tests if F achieved statistical significance (*P* < 0.05) and there was no significant variance in homogeneity. Data were presented as the mean ± SD, and *P* < 0.05 was considered statistically significant.

## Results and discussion

### Preparation and characterization of CHNQD-01281

Cinnamic acid and the pyridine ring possess a broad spectrum of biological and pharmacological properties, such as antitumor, antibacterial, and antioxidant activities (Lan et al. 2017; Ruwizhi et al. 2020). They are key intermediate products and present in many medicines. The introduction of cinnamic acid and a pyridine ring into the structure can help reduce toxic side effects and improve the lipid-water partition coefficient. Thus, we incorporated pyridine cinnamic acid into BFA in the hope of enhancing its antitumor effect and improving its druggability.

Using BFA as a starting material, **CHNQD-01281** was prepared as outlined in Fig. 1B. In detail, the 7-OH group of BFA was first protected with *tert*-butyldimethylsilyl chloride (TBSCl) in the presence of imidazole. The intermediate 7-TBS-CHNQD-01281 was prepared from the resulting 7-TBS-BFA by esterification with 3-(pyridin-3-yl)acrylic acid in the presence of 4-dimethylaminopyridine (DMAP) and 1-ethyl-3-(3-dimethylaminopropyl) carbodiimide hydrochloride (EDCl). Standard deprotection using TBAF·3H_2_O then furnished **CHNQD-01281** in a reasonable yield. The structure of the target was further characterized by NMR, HRESIMS data and single-crystal X-ray diffraction analysis (Supplementary Figs. S6–S8, Fig. 1A).

### Evaluation of CHNQD-01281 on a wide range of cancer cell lines

**Fig. 2:**
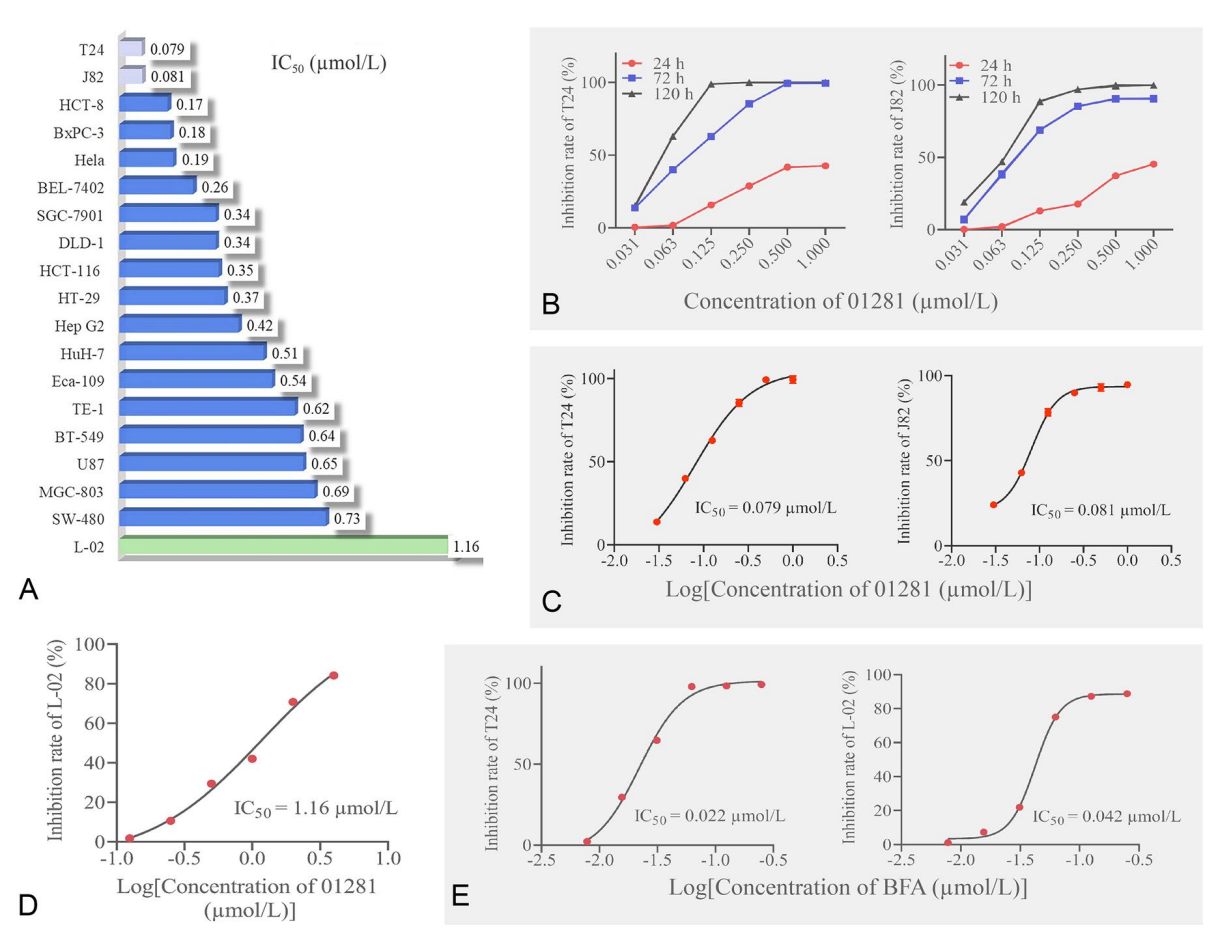
Evaluation of **CHNQD-01281** on a wide range of cancer cell lines. **A** Cell lines were treated with **CHNQD-01281** (0.031−1 μmol/L), inhibition rates were determined, and IC_50_ values (72 h) were conducted for a variety of cell lines. **B** T24 and J82 cell lines were treated for 24, 72 and 120 h with varying concentrations of **CHNQD-01281**. **C** IC_50_ values for 72 h in T24 and J82 cell lines treated with **CHNQD-01281**. **D** IC_50_ values for 72 h in L-02 cell line treated with **CHNQD-01281**. **E** T24 and L-02 cell lines were treated for 72 h with varying concentrations of BFA (0.0156−0.5 μmol/L), and IC_50_ values were calculated

To investigate whether **CHNQD-01281** had selective inhibitory effect on cancer cell lines, a variety of human tumor cell lines were assayed by MTT, such as bladder cancer cell lines (J82 and T24), colorectal cancer cell lines (HCT-116, HT-29, HCT-8, DLD-1 and SW-480), cervical cancer cell line (Hela), esophageal cancer cell lines (TE-1 and Eca-109), breast cancer cell line (BT-549), gastric carcinoma cell lines (MGC-803 and SGC-7901), glioma cell line (U87), pancreatic carcinoma cell line (BxPC-3) and hepatocellular carcinoma cell lines (Hep G2, BEL-7402 and HuH-7) (Fig. 2A). As shown in Table S1, **CHNQD-01281** exhibited potent antiproliferative activities on the above human cancer cell lines with IC_50_ values spanning the sub-micromolar to nanomolar range. It should be noted that **CHNQD-01281** dramatically inhibited the proliferation of T24 and J82 cell lines with IC_50_ values of 0.079 and 0.081 µmol/L, respectively, and IC_90_ values of 0.30 and 0.40 µmol/L, respectively.

Thus, the bladder cancer cell lines J82 and T24 were further investigated and treated by **CHNQD-01281** under gradient concentrations (0.031−1.00 µmol/L) and times (24, 72 and 120 h) to measure proliferation activities (Fig. 2B). However, the inhibition of T24 or J82 cell lines by **CHNQD-01281** was less than 50% even at the highest concentration of 1 µmol/L for 24 h. When exposed for 120 h, **CHNQD-01281** inhibited the growth of T24 by nearly 100% at 0.125 µmol/L. The data suggested that **CHNQD-01281** inhibited the proliferation of both T24 and J82 cells in a dose and time dependent manner, an exposure to **CHNQD-01281** for at least 72 and up to 120 h may be necessary to manifest sustained antiproliferative effects in bladder cancer cell lines. In particular, the IC_50_ value of **CHNQD-01281** against the human hepatocyte cell line L-02 cell line was 1.16 µmol/L (Fig. 2D), compared with the T24 or J82 cell lines (Fig. 2C), thus, the selectivity index (SI) was 14.68 and 14.32, respectively. As for BFA, the IC_50_ values of T24 and L-02 were 0.022 and 0.042, *µ*mol/L respectively (Fig. 2E), and the SI was only 1.91. Therefore, **CHNQD-01281** has a higher selectivity index compared to BFA.

### CHNQD-01281 inhibited colony formation and cell metastasis of bladder cancer cell lines

**Fig. 3:**
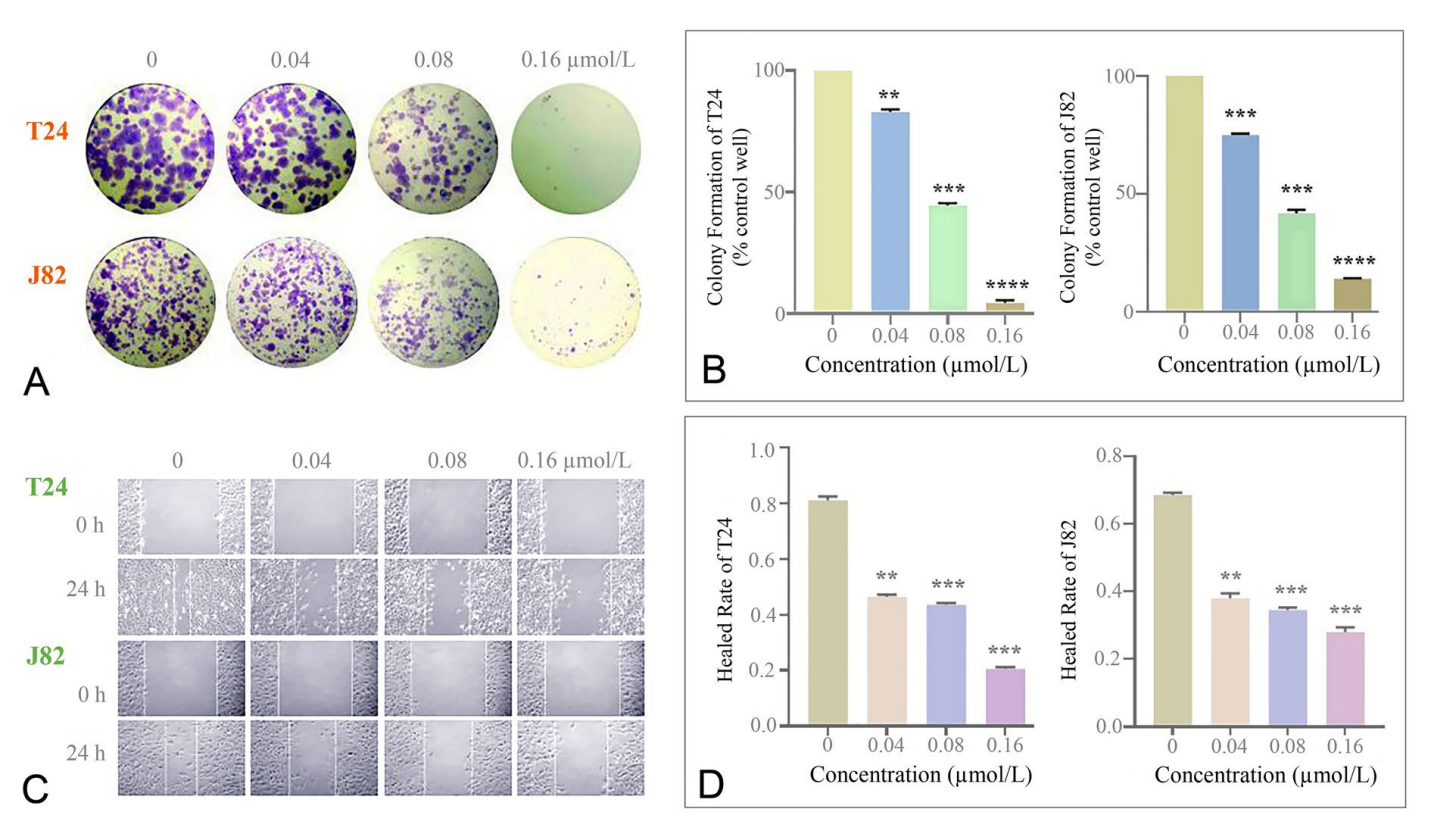
Effects of **CHNQD-01281** on colony formation and migration of T24 and J82 cell lines. **A** T24 and J82 cell lines were cultured for 12 days with varying concentrations of **CHNQD-01281** (0−0.16 μmol/L) and colonies were fixed and dyed with 0.1% crystal violet. **B** The relative colony numbers are shown. **C** Scratches of T24 and J82 cell colonies were generated with pipette tips (200 μL). After 24 h of incubation with 0−0.16 μmol/L **CHNQD-01281**, representative images were captured. **D** Histograms display the healed rate. Data are represented as the mean ± SD of three independent experiments. **P < 0.01, ***P < 0.001 and ****P < 0.0001 versus the 0 μmol/L group

Cell proliferation ability was also detected by colony formation assay (Kabakov et al. 2018), and the results are shown in Fig. 3A−B. **CHNQD-01281** was applied for 12 days to detect long-term proliferation inhibition of T24 and J82 cell lines. Compared with untreated cells, **CHNQD-01281** treated cells showed a dose-dependent effect on the inhibition of colony formation. At different concentrations of 0.04, 0.08 and 0.16 µmol/L, the inhibition rates of **CHNQD-01281** on colony formation were 16.97%, 52.52%, 95.54% versus 25.07% in the T24 cell line and 58.30%, 85.90% in J82 cell line, respectively.

Metastasis and invasiveness are important indications for progression of bladder cancer. To confirm whether **CHNQD-01281** could inhibit cancer metastasis, a wound healing assay was conducted as shown in Fig. 3C–D. Control cells without treatment obviously minimized the gap that was initially scraped after 24 h, while treatment of T24 and J82 cell lines with **CHNQD-01281** significantly inhibited migration in a dose-dependent manner. At the concentration of 0.16 µmol/L, the healed rate of T24 and J82 was 21% and 29%, respectively. Thus, **CHNQD-01281** markedly suppressed cell migration of T24 and J82 cell lines.

### CHNQD-01281 blocked cell cycle and induced apoptosis in bladder cancer cell lines

In normal cells, there is a dynamic balance between cell proliferation, division and cell death. Cell cycle represents a series of strictly-controlled events that is fundamental to cell proliferation (Liu et al. 2022; Nurse 2000). It is one of the characteristics of cancer that the severe imbalance of cell cycle makes tumor cells have the ability to proliferate indefinitely (Hanahan 2022). To block the abnormal progression of cell cycle, inhibiting the proliferation of cancer cell lines and promoting apoptosis are the basic strategy of cancer therapy.

**Fig. 4:**
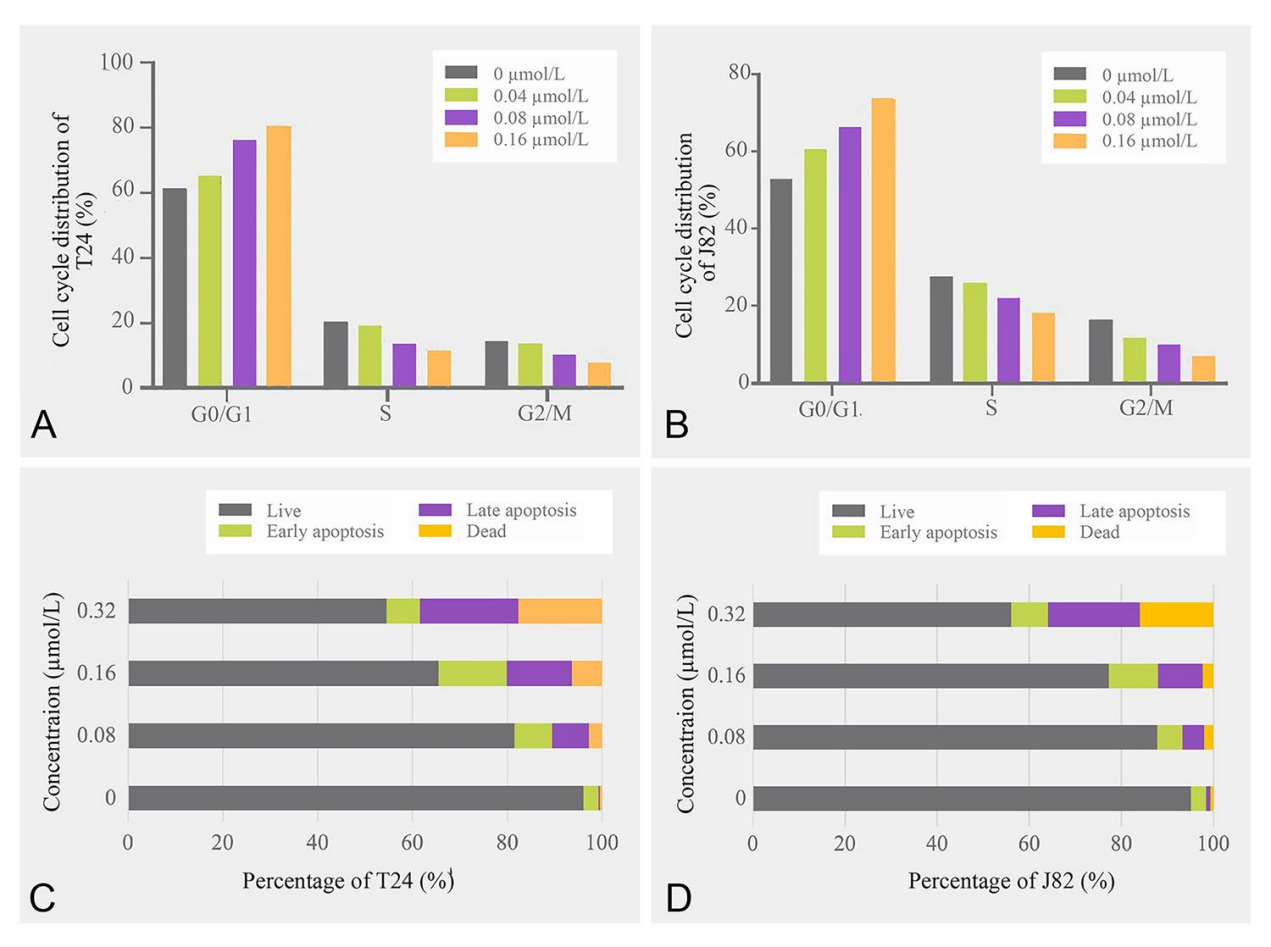
**CHNQD-01281** induced cell cycle arrest and cell apoptosis in T24 and J82 cell lines. **A** T24 and **B** J82 cell lines were incubated with varying concentrations of **CHNQD-01281** (0−0.16 μmol/L) and then analyzed by flow cytometry. Histograms display the cell cycle distribution percentages. Cell apoptosis was determined by Annexin V/PI staining. Cell population of different concentrations of **CHNQD-01281** in **C** T24 and **D** J82 cell lines

As shown, when concentrations of **CHNQD-01281** were 0.04, 0.08 and 0.16 µmol/L, the number of G0/G1 cells increased in a dose-dependent manner, from 61.4% to 65.0%, 75.2% and 80.6% in the T24 cell line and from 53.1% to 60.3%, 66.3% and 73.6% in the J82 cell line (Supplementary Fig. S9, Fig. 4A–B). However, the proportion of cells in S phase decreased significantly in a dose-dependent manner in T24 (20.5%, 18.9%, 13.5% and 11.4%) and J82 (27.5%, 26.0%, 22.1% and 18.2%). These results indicated that **CHNQD-01281** arrested cell cycle at G0/G1 phase. The G1/S transition is a key checkpoint in the cell cycle that regulates DNA replication. As the cell cycle progresses, G1 phase arrest leads to apoptosis or repair mechanisms.

Furthermore, treatment with **CHNQD-01281** induced a dose-dependent increase in the total apoptosis of T24 and J82 cell lines. Compared with the control group, the total number of apoptotic cells increased from 3.45% to 27.8% in T24 and from 4.15% to 27.9% in J82 at the highest **CHNQD-01281** concentration of 0.32 µmol/L (Supplementary Fig. S10, Fig. 4C–D). Meanwhile, the expression of apoptosis-related proteins cleaved PARP and cleaved caspase-3 were detected by Western blot. As shown in Fig. S11b, the expression of both cleaved PARP and cleaved caspase-3 increased significantly after **CHNQD-01281** treatment, with the maximum expression level of cleaved caspase-3 being 1.23-fold and the maximum expression level of cleaved PARP being 0.95-fold of the control group. Therefore, **CHNQD-01281** can induce DNA damage to arrest the cell cycle at the G0/G1 phase, affect the expression of DNA damage repair enzyme PARP, and finally induce apoptosis of bladder cancer cell lines.

### Optimization of the dissolution system of CHNQD-01281

The problem of poor water solubility is one of the factors that seriously restricts the preclinical application of BFA. Therefore, from the perspective of pharmaceutics, it is of great application value to explore safe and effective methods to improve its solubility. Based on the previous screening of commonly used solvents, solubilizers and co-solvents for BFA and its derivatives, we explored the solubility of **CHNQD-01281**, which was compared and assessed in selected aqueous solutions (Table 1). Saline or PBS were commonly used solvents, but the solubility of BFA or **CHNQD-01281** was less than 1 mg/mL when used alone. No significant improvement in solubility was observed after the addition of DMSO or PEG400. As shown, the solubility of **CHNQD-01281** was 12–15 mg/mL in saline solution containing 5% DMSO and 10% solutol HS-15, while the solubility of BFA was only 1–1.5 mg/mL. The data suggest that the introduction of the pyridine cinnamic acid into BFA can effectively improve the solubility of **CHNQD-01281** approximately tenfold. More importantly, the solution system of **CHNQD-01281** with saline/DMSO/solutol HS-15 (85/5/10) can fully meet the needs of follow-up experiments in vivo, such as pharmacodynamics, pharmacokinetics and safety evaluation.

**Table 1:** Solubility of **CHNQD-01281**

No.	Solvent composition			Volume	Solubility (mg/mL)	
	Aqueous solution	Solubilizer	Surfactant		BFA	**CHNQD-01281**
1	Saline^a^	–	–	100	< 1	< 1
2	Saline	DMSO	–	95/5	< 1	< 1
3	PBS^b^	DMSO	–	95/5	< 1	< 1
4	Saline	PEG400^c^	–	95/5	< 1	< 1
5	Saline	PEG400	–	80/20	< 1	< 1
6	Saline	DMSO	solutol HS-15^d^	85/5/10	1–1.5	12–15
7	Saline	–	solutol HS-15	95/5	< 1	< 1
8	Saline	–	solutol HS-15	90/10	< 1	< 1

^a^Saline, 0.9% NaCl

^b^PBS, phosphate-buffered saline, pH 7.35–7.45

^c^PEG400, poly (ethylene glycol)

^d^Solutol HS-15, poly (ethylene glycol) 12-hydroxystearate. The data were detected at pH 7.4, 25 °C

### CHNQD-01281 suppressed bladder cancer growth in vivo

**Fig. 5:**
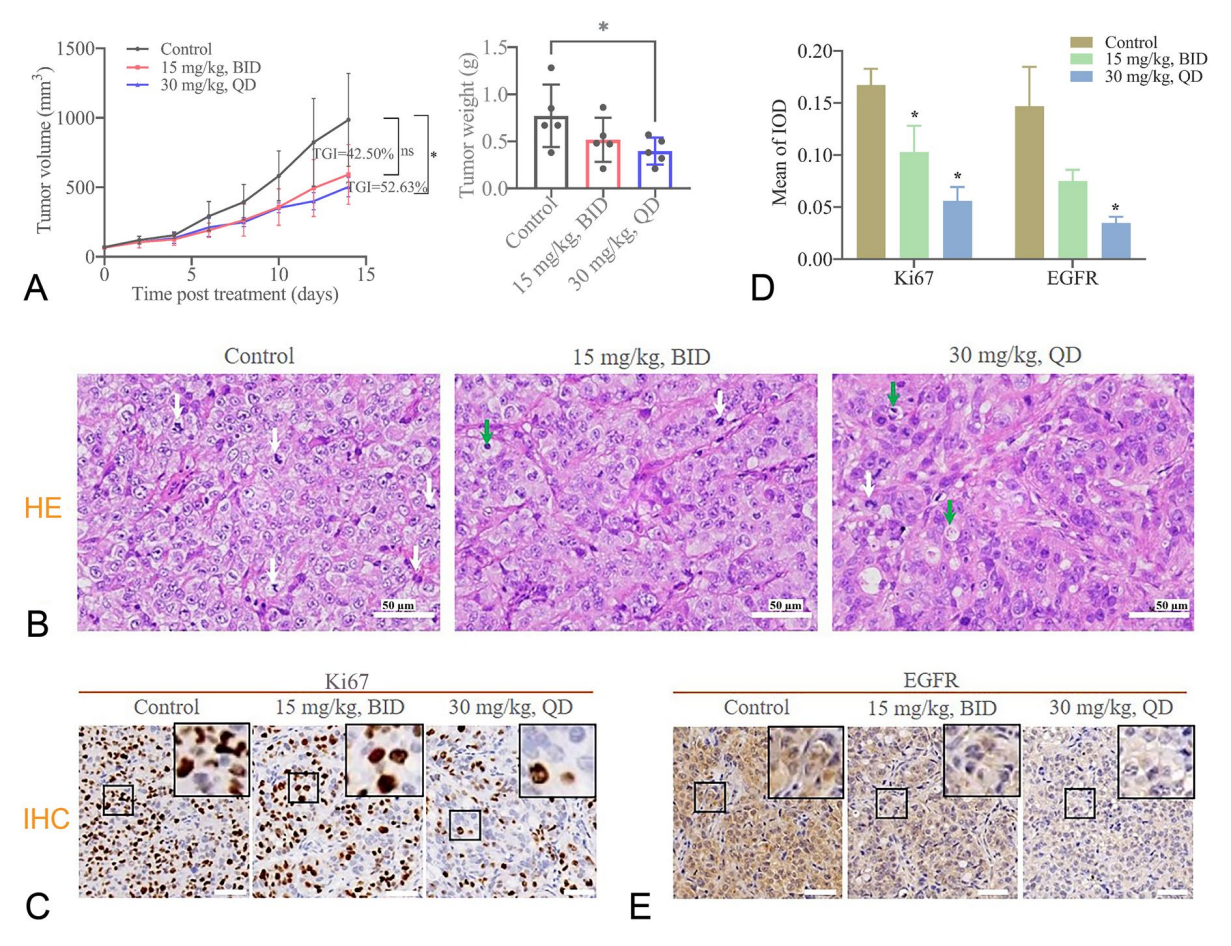
**CHNQD-01281** reduced the tumor burden in a T24 xenograft mouse model. **A** Tumor volume changes during treatment and weight of the excised tumors of each group (*n* = 5). **B** HE staining of tumor tissues. The nuclei with hyperchromatism and pleomorphism (white arrow), necrotic and apoptotic tumor cells (green arrow) were indicated. **C** Ki67 and EGFR (**E**) were analyzed by immunohistochemical staining. **D** Quantification of Ki67 and EGFR protein expression. Data presented as mean ± SD. *P < 0.05 versus control. Scale bar indicates 50 μm

The in vitro study showed that **CHNQD-01281** significantly inhibited proliferation and migration and induced apoptosis of T24 and J82 cell lines. Therefore, xenograft and homologous tumor models were constructed to further evaluate the antitumor effect of **CHNQD-01281** in vivo. The mouse bladder cancer xenograft model was established by the subcutaneous inoculation of the T24 cell line, then **CHNQD-01281** was intraperitoneally administrated for two consecutive weeks (Fig. 5A). As expected, the tumor tissues were significantly shrunk in **CHNQD-01281**-treated groups compared with the control group. In addition, the tumor volumes in the 30 mg/kg (i.p., QD) **CHNQD-01281**-treated group were dramatically smaller than those in the control group, with a tumor growth inhibition rate (TGI %) value of 52.63%, which was superior to 15 mg/kg group (i.p., BID). From the data of tumor weight, the 30 mg/kg group was reduced by 48.57%, significantly lower than that of the control group. Hematoxylin–eosin (HE) staining showed that the control group had more mitotic figures and apparent strange type nuclei, while in the **CHNQD-01281** treatment group, the tumor cells were arranged regularly, and the nuclear pyknosis and lysis were observed (Fig. 5B). Immunohistochemical results showed that compared with the control group, the 15 and 30 mg/kg groups reduced the expression level of Ki67 by 38.32% and 66.47%, respectively (Fig. 5C–D).

**Fig. 6:**
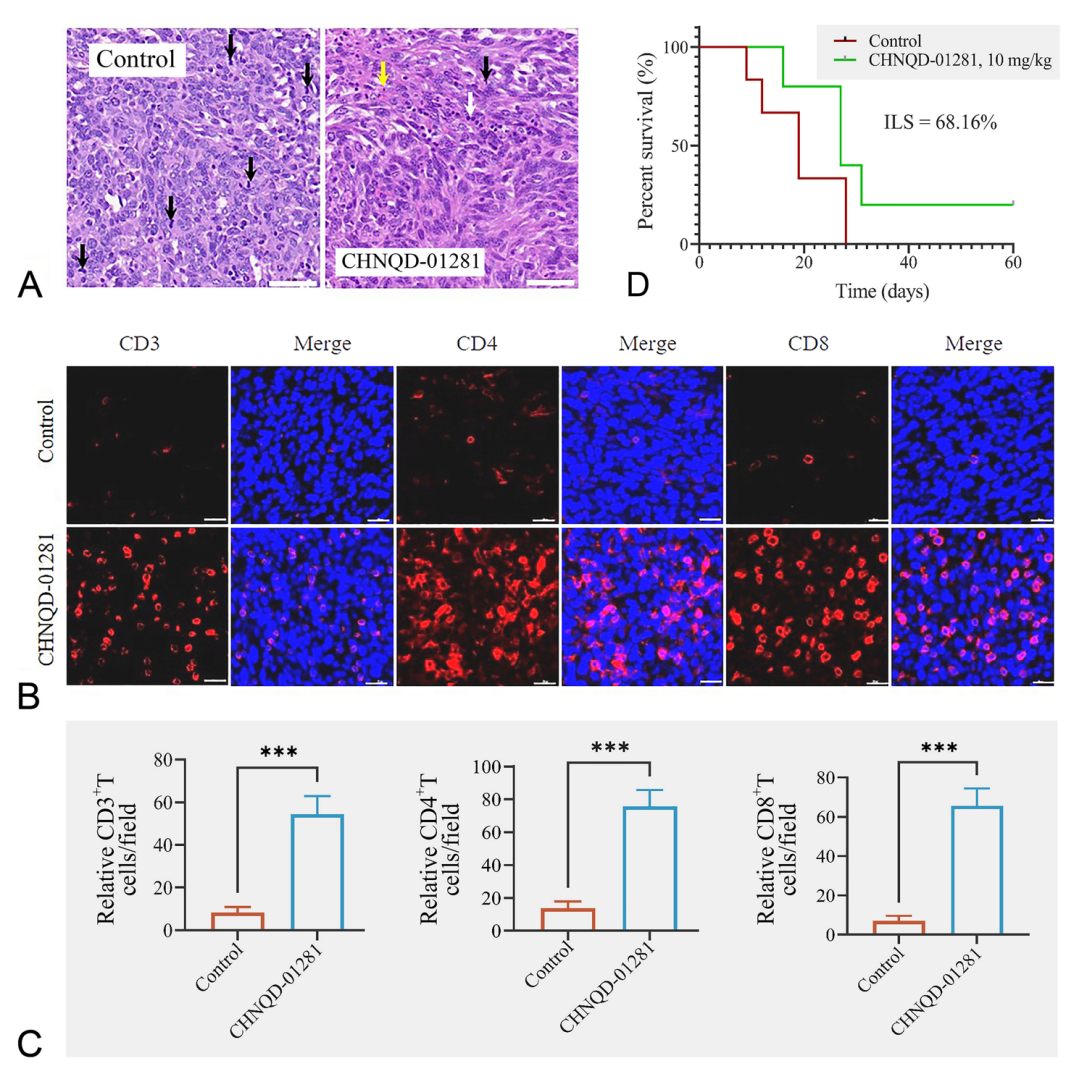
**CHNQD-01281** prolonged the survival time in an MB49 homologous tumor model. **A** HE staining of tumor tissue. Mitotic figures (black arrow), eosinophilic staining (yellow arrow) and lymphocytes infiltration (white arrow) are indicated. Scale bar indicates 50 μm. CD3^+^, CD4^+^ and CD8^+^T cells were analyzed by **B** immunofluorescence staining and **C** quantitative analysis. Data presented as mean ± SD. ***P < 0.001 versus control. Scale bar indicates 20 μm. **D** Survival curve of MB49 homologous tumor mice model treated by intratumoral injection with either the vehicle (DMSO) or **CHNQD-01281** (*n* = 5)

In the MB49 homologous tumor model, HE staining showed that mitotic figures were common in the control group, while it was rare in the **CHNQD-01281** treatment group (10 mg/kg, intratumoral injection, QD) (Fig. 6A). In addition, apoptosis, necrosis, and increased eosinophil cytoplasm were observed and there was a large number of lymphocyte infiltration in the treatment group. Immunofluorescence staining showed that the infiltration of CD3^+^, CD4^+^ and CD8^+^T cells in the treatment group was significantly increased (Fig. 6B–C). Importantly, the survival time of the tumor-bearing mice was significantly prolonged by intratumoral administration of **CHNQD-01281**, and the life prolongation rate was 68.16% (Fig. 6D). In addition, 20% of the mice in the treated group had tumor disappearance, and the tumor did not recur within 30 days after the cessation of drug administration. These results suggest that **CHNQD-01281** promoted the infiltration of T lymphocytes in the tumor tissues of MB49 tumor-bearing mice and induced antitumor immunity, thus, achieving the purpose of tumor treatment and prevention.

### Effects of CHNQD-01281 on EGFR/PI3K/AKT and EGFR/ERK pathways

EGFR is overexpressed in malignant urothelial cells, which is associated with the deterioration, invasiveness and metastasis of tumors. It is a potential therapeutic target for bladder carcinoma. Therefore, we examined the expression of EGFR and its downstream proteins after treating bladder cancer cells with **CHNQD-01281** (Supplementary Fig. S11). It showed that **CHNQD-01281** significantly downregulated EGFR expression in T24 and J82 cell lines compared with the control group, with maximum down-regulation levels of 77.61% and 87.61% at a concentration of 0.16 µmol/L, respectively. The expression of EGFR was significantly down-regulated in a dose-dependent manner, suggesting that EGFR is involved in the inhibitory effect of **CHNQD-01281** on bladder cancer cell proliferation. The expression levels of *p*-ERK/ERK, *p*-AKT/AKT, and p-mTOR/mTOR were decreased by 96.95%, 99.96% and 60.36%, respectively, at the dose of 0.16 µmol/L. Immunohistochemical staining revealed that the expression of EGFR in tumor-bearing mice treated with **CHNQD-01281** showed a downward trend, and the 30 mg/kg dose group had a significantly down-regulated expression of EGFR (Fig. 5E). Taken together, **CHNQD-01281** inhibited the proliferation of bladder cancer cells and induced apoptosis through EGFR/PI3K/AKT and EGFR/ERK pathways to exert antitumor effects.

### Transcriptome analysis identifies the differential expressed genes

**Fig. 7:**
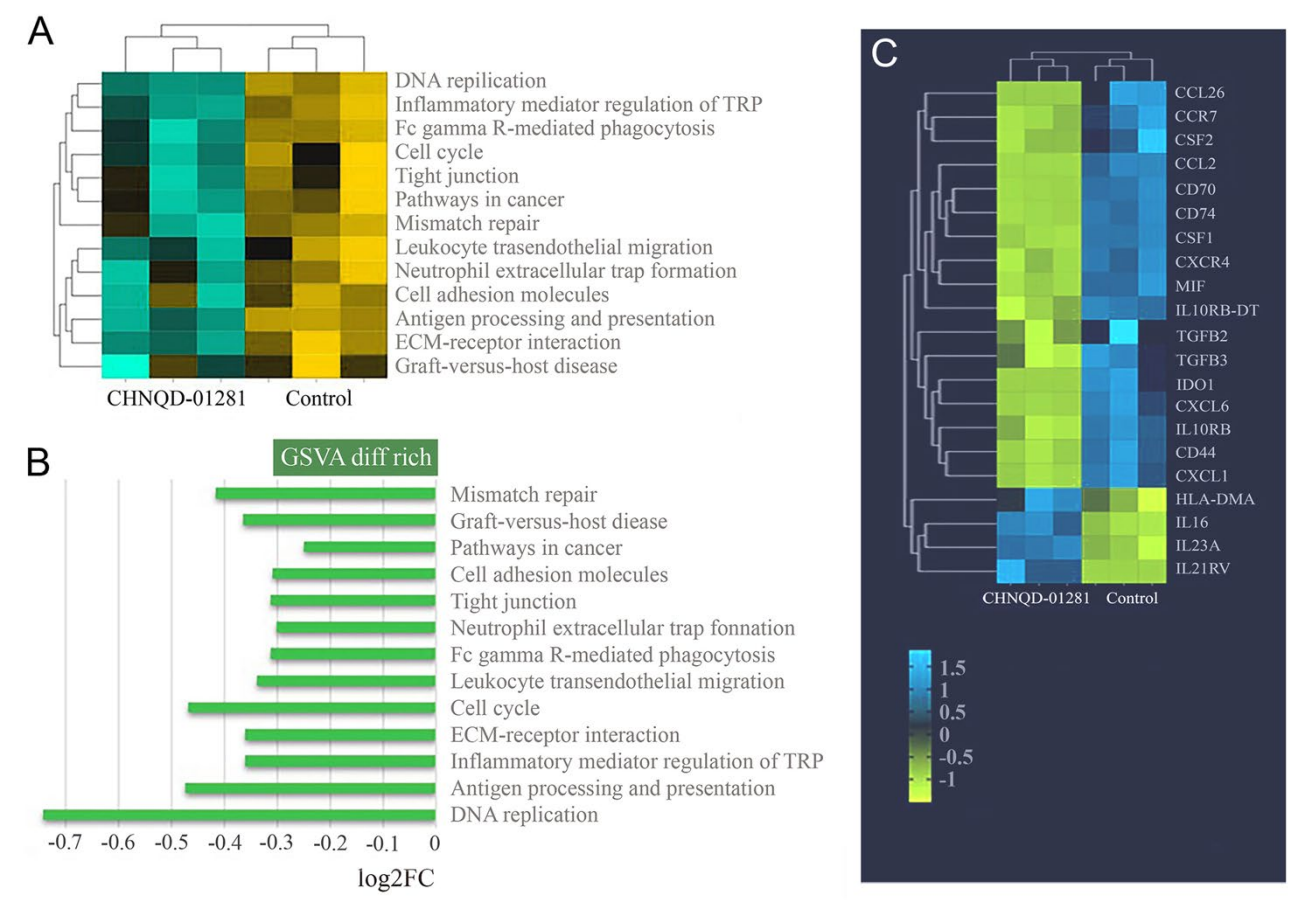
Transcriptome analysis of **CHNQD-01281** treated T24 cell line. **A** Heatmap and **B** differential enrichment analysis of gene set variation analysis (GSVA) was performed to display the differences in gene sets among each sample (|log2FC|≥ 0.25, *P*_*adjust*_ < 0.05). **C** Differential gene expression analysis of immunomodulatory factors and chemokines (fold change ≥ 2, *P* < 0.01)

To further explore the antitumor mechanism of **CHNQD-01281**, a transcriptome analysis was conducted. As shown in Fig. S12a–c, there were about 2231 upregulated genes and 2664 downregulated genes identified (fold change ≥ 2 and *P* < 0.001) in the **CHNQD-01281** treated group compared with the control group (CON). GO and KEGG enrichment analysis showed that **CHNQD-01281** had a significant inhibitory effect on some biological processes, such as double-strand break repair via break-induced replication, DNA strand elongation involved in DNA replication, protein exit from endoplasmic reticulum and cell cycle (Supplementary Fig. S12d–e). Both gene set variation analysis and gene set enrichment analysis also revealed that the **CHNQD-01281** treated group had suppressed genes related to cell cycle, DNA replication and mismatch repair (Fig. 7A–B, Supplementary Fig. S13). Moreover, differential gene expression analysis showed that **CHNQD-01281** reduced the gene expression of immunosuppressive cytokines and chemokines (Leone et al. 2020; Wang et al. 2022c), including IDO1, CSF1, CSF2, TGFβ2, TGFβ3, CCL2, CCL26, CXCL1, CD70, CD74 and CD44 but increased the gene expression of cytokines that function as antitumor immune modulators (Xu et al. 2018), including HLA-DMA, IL16, IL23A and IL21R (Fig. 7C).

### Tissue distribution and safety profile of CHNQD-01281 in vivo

To further investigate the safety profile of **CHNQD-01281** in vivo, the HE staining of the heart, liver, spleen, lung, kidney and duodenum of C57 mice were analyzed after the single dose of **CHNQD-01281** (100 mg/kg, i.p.). As shown in Fig. S14, there were no obvious abnormalities in cell and tissue morphology in the **CHNQD-01281** treated group. These results suggested that there was no significant toxicity at the current dose of **CHNQD-01281**. The distribution of **CHNQD-01281** in tumor and main organs of C57BL/6 mice was then analyzed. The concentrations of **CHNQD-01281** in tumor, heart, duodenum and spleen were higher than the other tissues within 0.5 h; a rapid concentration elimination was also detected within 1 h in these organs, suggesting the rapid elimination of **CHNQD-01281** in vivo*.*

## Conclusion

In the present work, **CHNQD-01281**, a brefeldin A-pyridylacrylic acid derivative, was found to exhibit excellent selective anticancer activities against bladder cancer in vitro and in vivo, as further demonstrated through multiple mechanisms, including double-strand DNA break repair via break-induced replication, DNA strand elongation involved in DNA replication, protein exit from endoplasmic reticulum, mismatch repair, cell cycle and apoptosis. These properties overlap in part with BFA's classical antitumor mechanisms, such as blocking protein transport between the endoplasmic reticulum and the Golgi apparatus, cell cycle, and interfering with cell division (Lu et al. 2022; Zhu et al. 2017). The frequency of mutations in the DNA-damage repair (DDR) gene was higher in MIBC than that in NMIBC (91% vs. 78%), suggesting the possibility of treating advanced bladder cancer with PARP inhibitors (Wang et al. 2022b). The activation of the MAPK and PI3K/mTOR signaling pathways were reported in about 70% of bladder tumors, including amplification of EGFR (9%) (Felsenstein et al. 2018). Interestingly, we found that **CHNQD-01281** significantly down-regulated the expression of EGFR and PARP and inhibited the activation of PI3K/AKT/mTOR and EGFR/ERK pathways. In particular, **CHNQD-01281** can activate the expression of tumor-killing T cells by reducing inhibitory chemokines and activating immune regulatory factors to some extent. In addition, **CHNQD-01281** displayed high solubility and favorable safety.

**CHNQD-01281** exerted promising potential in the treatment of bladder cancer with high selectivity, improved solubility and favorable safety. As a lead candidate, **CHNQD-01281** is currently undergoing further characterization of the preclinical profile and full elucidation of the molecular mechanisms in follow-up studies.

### Supplementary Information

Below is the link to the electronic supplementary material.

Supplementary file 1 (DOCX 10654 kb)

## Data Availability

The data that supports the findings of this study are included in this published article (and its supplementary information file).
