# Supplementary material for: Discovery of a potential bladder cancer inhibitor CHNQD-01281 by regulating EGFR and promoting infiltration of cytotoxic T cells
Source: Mar Life Sci Technol. 2024 Aug 5;6(3):502–14. doi: 10.1007/s42995-024-00246-w (PMC11358582; doi:10.1007/s42995-024-00246-w)
Supplement: Supplementary file 1 — (DOCX 10654 kb) [file 42995_2024_246_MOESM1_ESM.docx]

**Discovery of a potential bladder cancer inhibitor CHNQD-01281 by regulating EGFR and promoting infiltration of cytotoxic T cells**

Jian-Yu Liu^1^ **·** Yao-Yao Jiang^1^ **·**Peng-Jie Li ^1^ **·** Bo Yao*^,2^ **·** Yi-Jing Song^1^ **·** Ji-Xiu Gao^1^ **·** Gulab Said^1,3^ **·** Yang Gao^1^ **·** Jun-Yu Lai^1^ **·** Chang-Lun Shao*^,1,4,5^

1 Key Laboratory of Marine Drugs, the Ministry of Education of China, School of Medicine and Pharmacy, Ocean University of China, Qingdao 266003, China

2 Department of Critical Care Medicine, the Affiliated Hospital of Qingdao University, Qingdao 266003, China

3 Department of Chemistry, Women University Swabi, Swabi 23430, Pakistan

4 Laoshan Laboratory, Qingdao 266237, China

5 Key Laboratory of Tropical Medicinal Resource Chemistry of Ministry of Education, College of Chemistry and Chemical Engineering, Hainan Normal University, Haikou 571158, China

* Correspondence: shaochanglun@163.com (Chang-Lun Shao); icuyaobo@126.com (Bo Yao)

Jian-Yu Liu and Yao-Yao Jiang contributed equally to this work.

**Content of Supporting Information**

**Fig. S1−S8**. Compound characterization spectra.

**Table S1**. Evaluation of compound **CHNQD-01281** on a wide range of cell lines.

**Fig. S9**. **CHNQD-01281** induced cell cycle arrest in T24 and J82 cell lines.

**Fig. S10**. **CHNQD-01281** induced cell apoptosis in T24 and J82 cell lines.

**Fig. S11**. Effects of **CHNQD-01281** on EGFR/PI3K/AKT and EGFR/ERK.

**Fig. S12**. Transcriptome analysis.

**Fig. S13**. Gene set enrichment analysis (GSEA) of **CHNQD-01281** treated T24 cell line.

**Fig. S14**. Safety profile and tissue distribution of **CHNQD-01281** *in vivo*.

**Fig. S15**. **CHNQD-01281** inhibited T24 xenograft mouse model *in vivo*.

**Compound Characterization Spectra**

**Brefeldin A (BFA)**: white, amorphous powder. ^1^H NMR (500 MHz, DMSO-*d*_6_) *δ* 7.34 (1H, dd, *J* = 15.5, 3.0 Hz, H-3), 5.75–5.60 (2H, overlapped, H-2, H-11), 5.20 (1H, dd, *J* = 15.2, 9.6 Hz, H-10), 5.10 (1H, s, 4-OH), 4.71 (1H, m, H-15), 4.48 (1H, s, 7-OH), 4.04 (1H, m, H-7), 3.92 (1H, d, *J* = 9.2 Hz, H-4), 2.30 (1H, m, H-9), 2.02–1.87 (2H, overlapped, H-8, H-12), 1.87–1.60 (6H, overlapped, H-5, H-6a, H-6b, H-12, H-13, H-14), 1.47 (1H, m, H-14), 1.30 (1H, m, H-8), 1.18 (3H, d, *J* = 6.3 Hz,16-CH_3_), 0.75 (1H, m, H-13); ^13^C NMR (125 MHz, DMSO-*d*_6_) *δ* 165.7 (C-1), 154.4 (C-3), 137.1 (C-10), 129.2 (C-11), 116.3 (C-2), 74.3 (C-4), 70.9 (C-15), 70.5 (C-7), 51.7 (C-5), 43.3 (C-9), 43.1 (C-8), 40.9 (C-6), 33.4 (C-14), 31.5 (C-12), 26.5 (C-13), 20.7 (C-16); ESIMS *m/z* 281.17 [M + H]^+^, 263.19 [M + H − H_2_O]^+^, 245.19 [M + H − 2H_2_O]^+^.


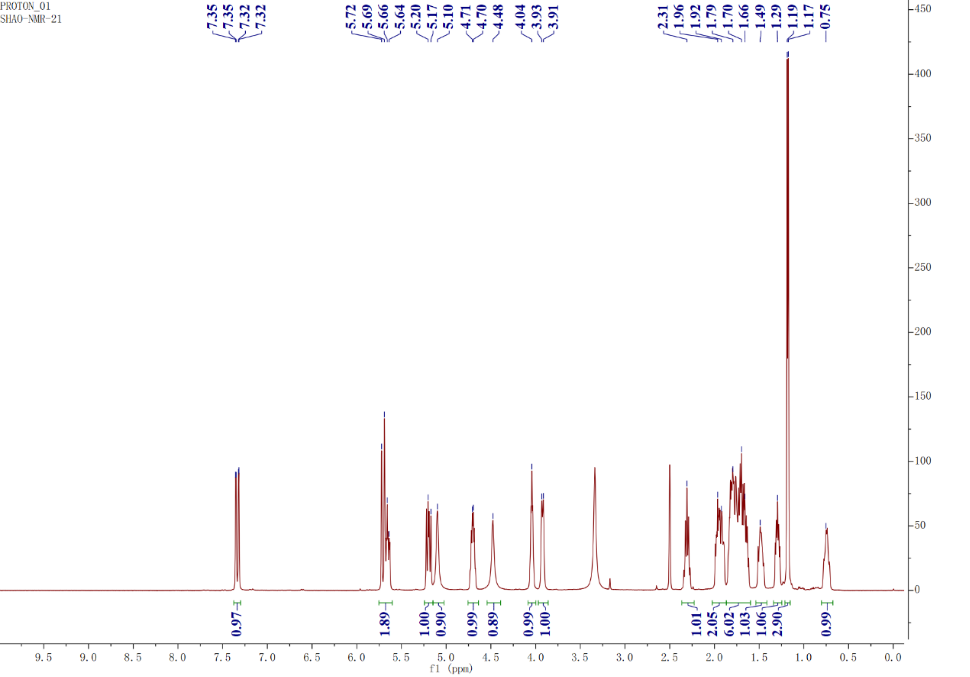


**Fig. S1.** ^1^H NMR (500 MHz, DMSO-*d*_6_) spectrum of BFA.


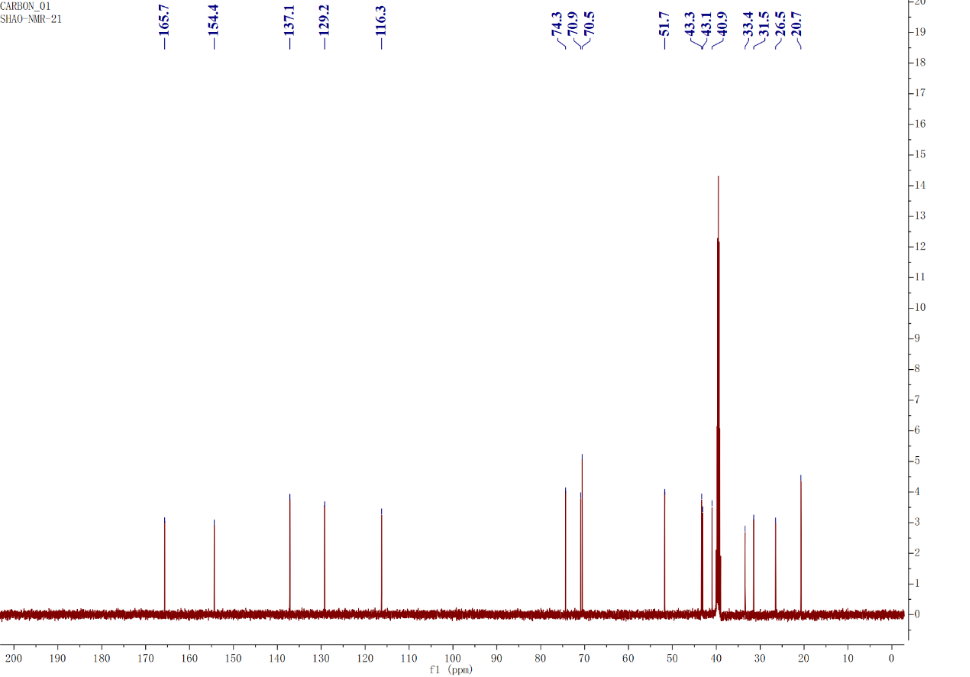
**Fig. S2.** ^13^C NMR (125 MHz, DMSO-*d*_6_) spectrum of BFA.


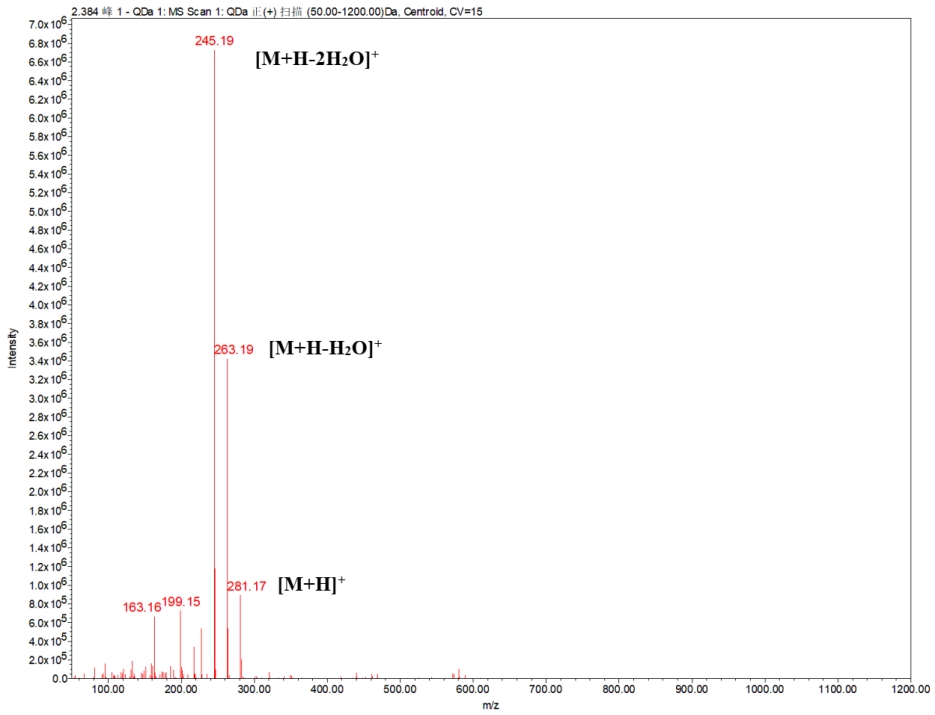


**Fig. S3.** ESIMS spectrum of BFA.


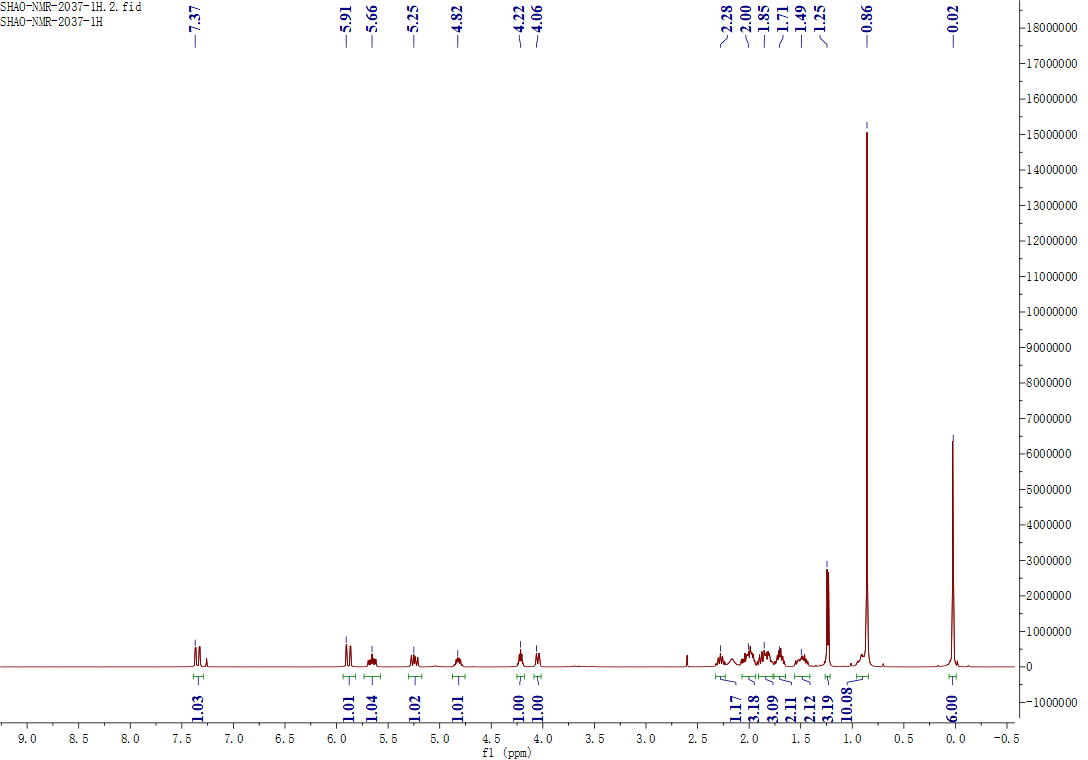


**Fig. S4.** ^1^H NMR (400 MHz, CDCl_3_) spectrum of compound **7-TBS-BFA**.

**Fig. S5.** ^13^C NMR (100 MHz, CDCl_3_) spectrum of compound **7-TBS-BFA**.


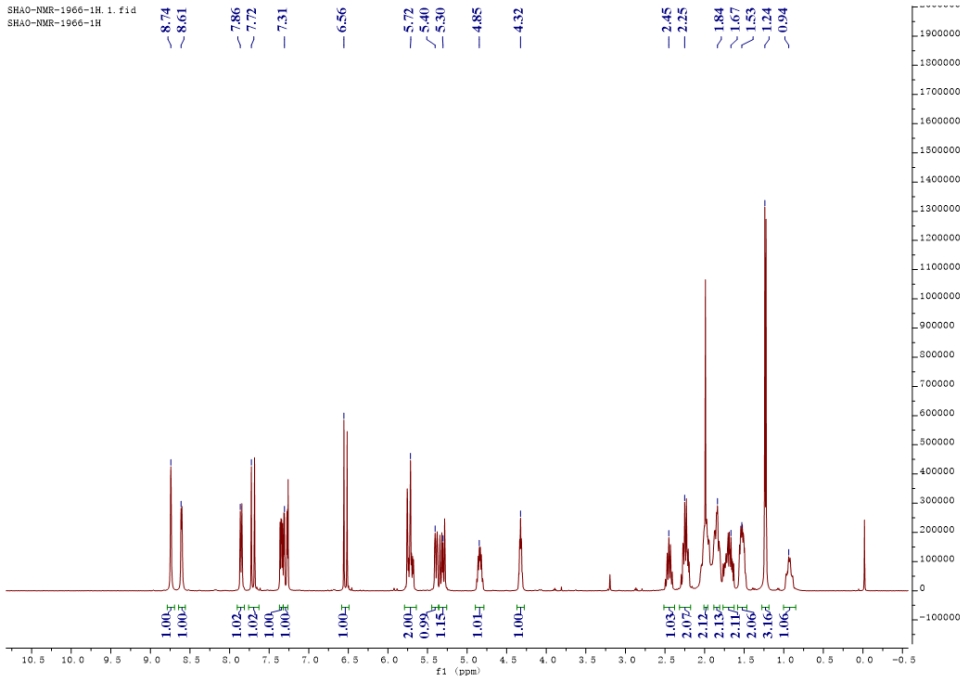


**Fig. S6.** ^1^H NMR (400 MHz, CDCl_3_) spectrum of compound **CHNQD-01281**.


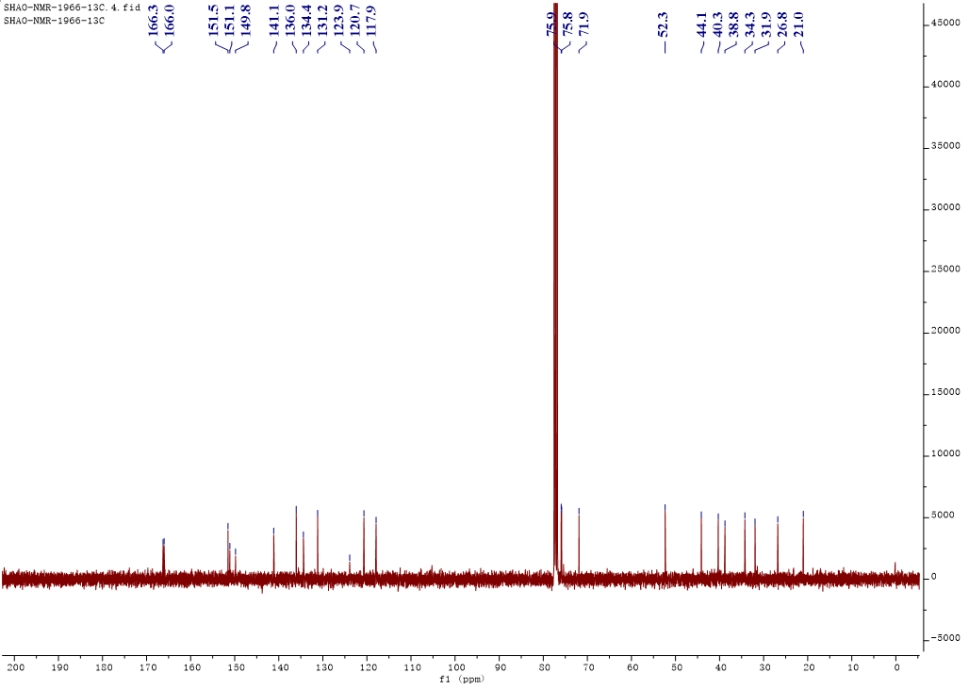


**Fig. S7**. ^13^C NMR (100 MHz, CDCl_3_) spectrum of compound **CHNQD-01281**.


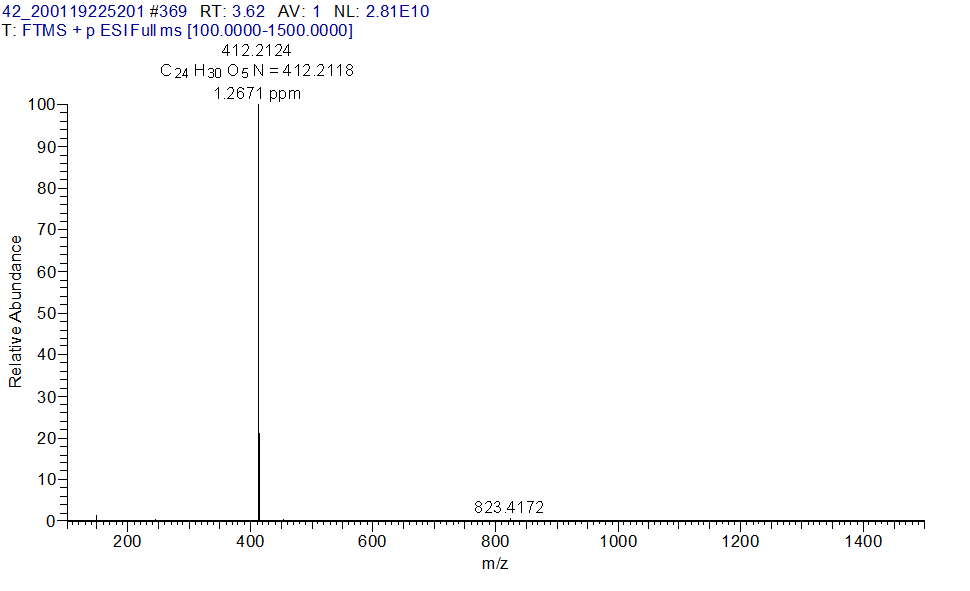


**Fig. S8**. HRESIMS spectrum of compound **CHNQD-01281**.

**Table S1** Evaluation of compound **CHNQD-01281** on a wide range of cell lines

| **Cell lines** | **IC_50_ (*µ*M) *^a^*** | **SI** | **IC_90_ (*****µ*M) *^a^*** |
| --- | --- | --- | --- |
| Hep G2 | 0.42 | 2.76 | ＞1 |
| **J82** | **0.081** | **14.32** | 0.40 |
| **T24** | **0.079** | **14.68** | 0.30 |
| Hela | 0.19 | 6.11 | 0.78 |
| BxPC-3 | 0.18 | 6.44 | 0.35 |
| HCT-116 | 0.35 | 3.31 | 0.64 |
| HT-29 | 0.37 | 3.14 | ＞1 |
| BEL-7402 | 0.26 | 4.46 | 0.82 |
| DLD-1 | 0.34 | 3.41 | 0.64 |
| BT-549 | 0.64 | 1.81 | ＞1 |
| HCT-8 | 0.17 | 6.82 | 0.44 |
| MGC-803 | 0.69 | 1.68 | ＞1 |
| HuH-7 | 0.51 | 2.27 | ＞1 |
| SGC-7901 | 0.34 | 3.41 | 0.75 |
| SW-480 | 0.73 | 1.59 | ＞1 |
| TE-1 | 0.62 | 1.87 | ＞1 |
| U87 | 0.65 | 1.78 | ＞1 |
| Eca-109 | 0.54 | 2.15 | ＞1 |
| **L-02** | **1.16** | **—** | **2.52** |

*^a^* Results were the average of three independent experiments, each performed in duplicate. Standard deviations were less than ± 10%.


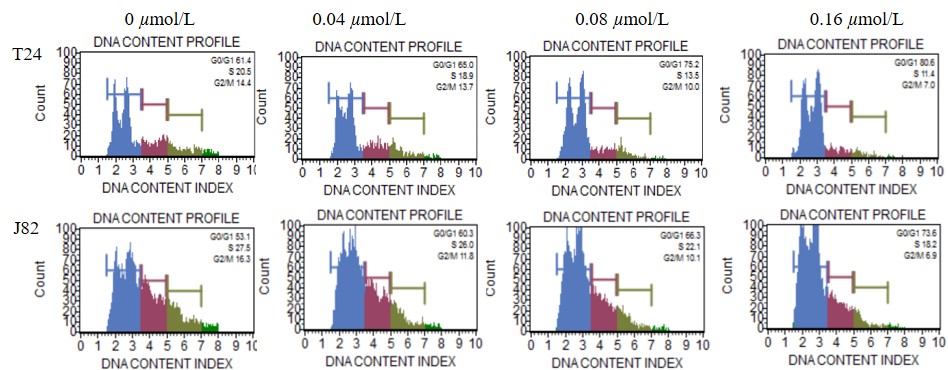


**Fig. S9. CHNQD-01281** induced cell cycle arrest in T24 and J82 cell lines.


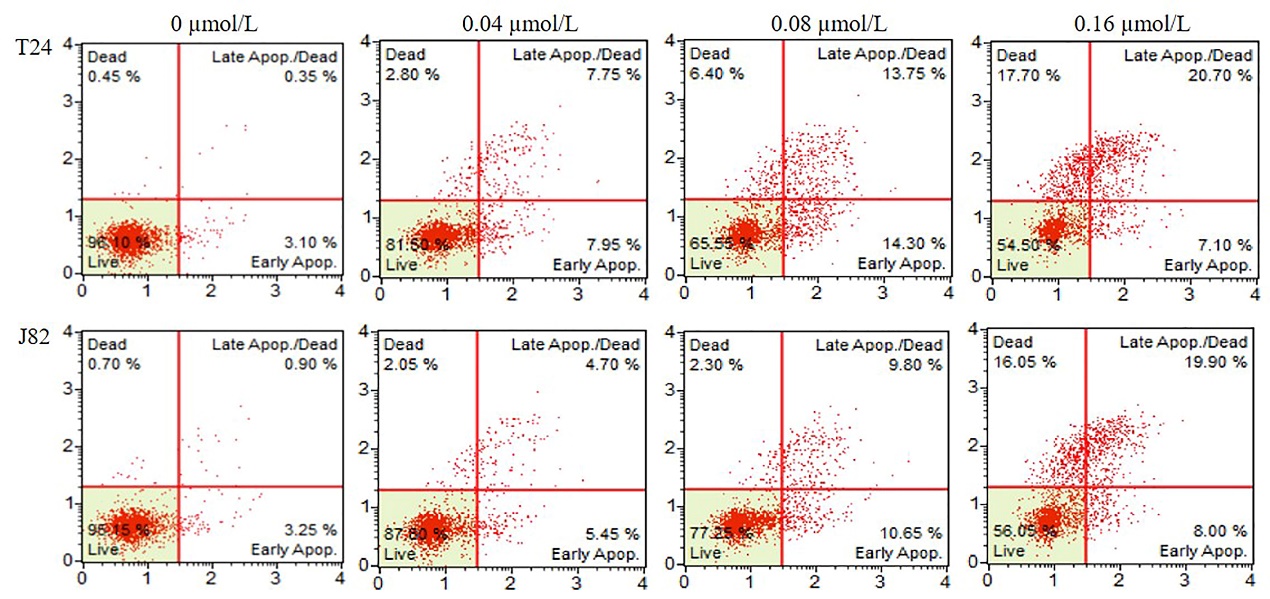


**Fig. S10. CHNQD-01281** induced cell apoptosis in T24 and J82 cell lines.


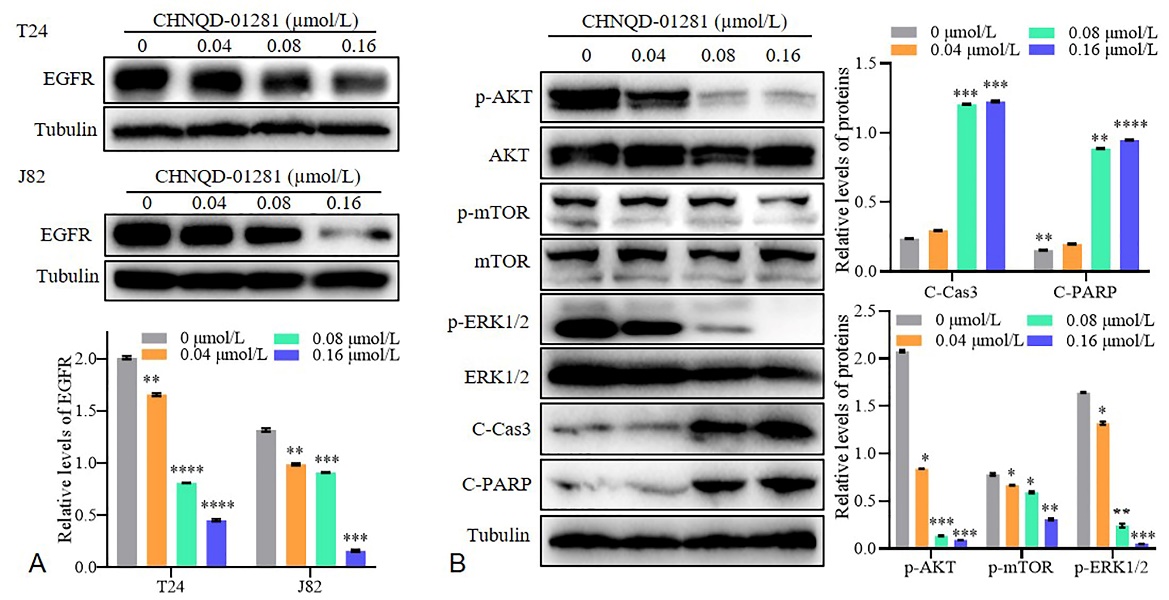


**Fig. S11.** Effects of **CHNQD-01281** on EGFR/PI3K/AKT and EGFR/ERK. **A** Western blot for EGFR in T24 and J82 cell lines. **B** Western blot analysis of AKT, p-AKT, mTOR, p-mTOR, ERK, p-ERK, C-Cas3 and C-PARP. Data are represented as the mean ± SD of three independent experiments. **P* < 0.05, *****P* < 0.01, ****P* < 0.001 and *****P*< 0.0001 vs the 0 *μ*mol/L.


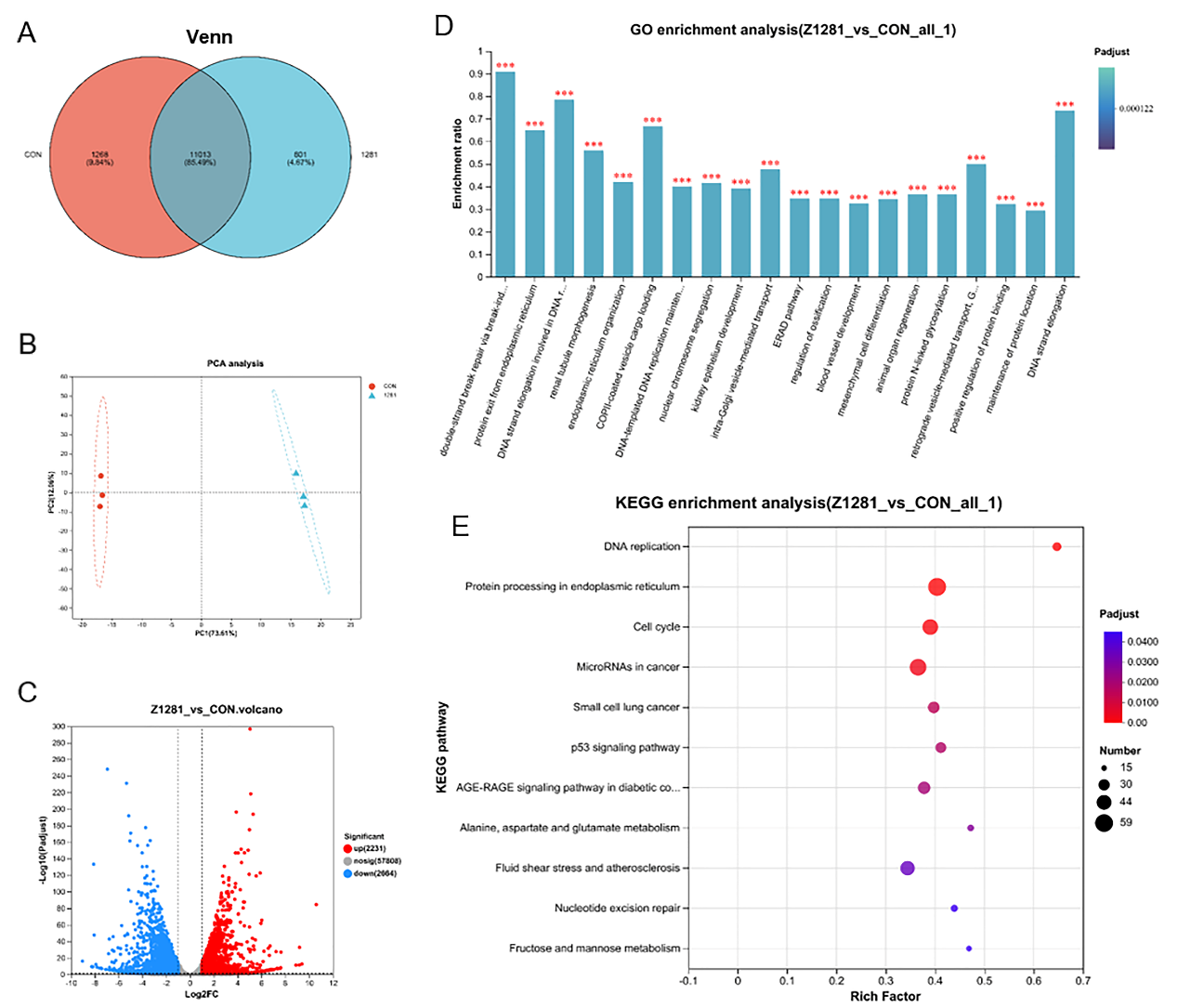


**Fig. S12.** Transcriptome analysis. **A** Venn diagram. **B** PCA analysis. **C** Volcano plot of differential expression gene. **D** GO enrichment analysis. **E** KEGG enrichment analysis. 1281 or Z1281 denotes **CHNQD-01281**.


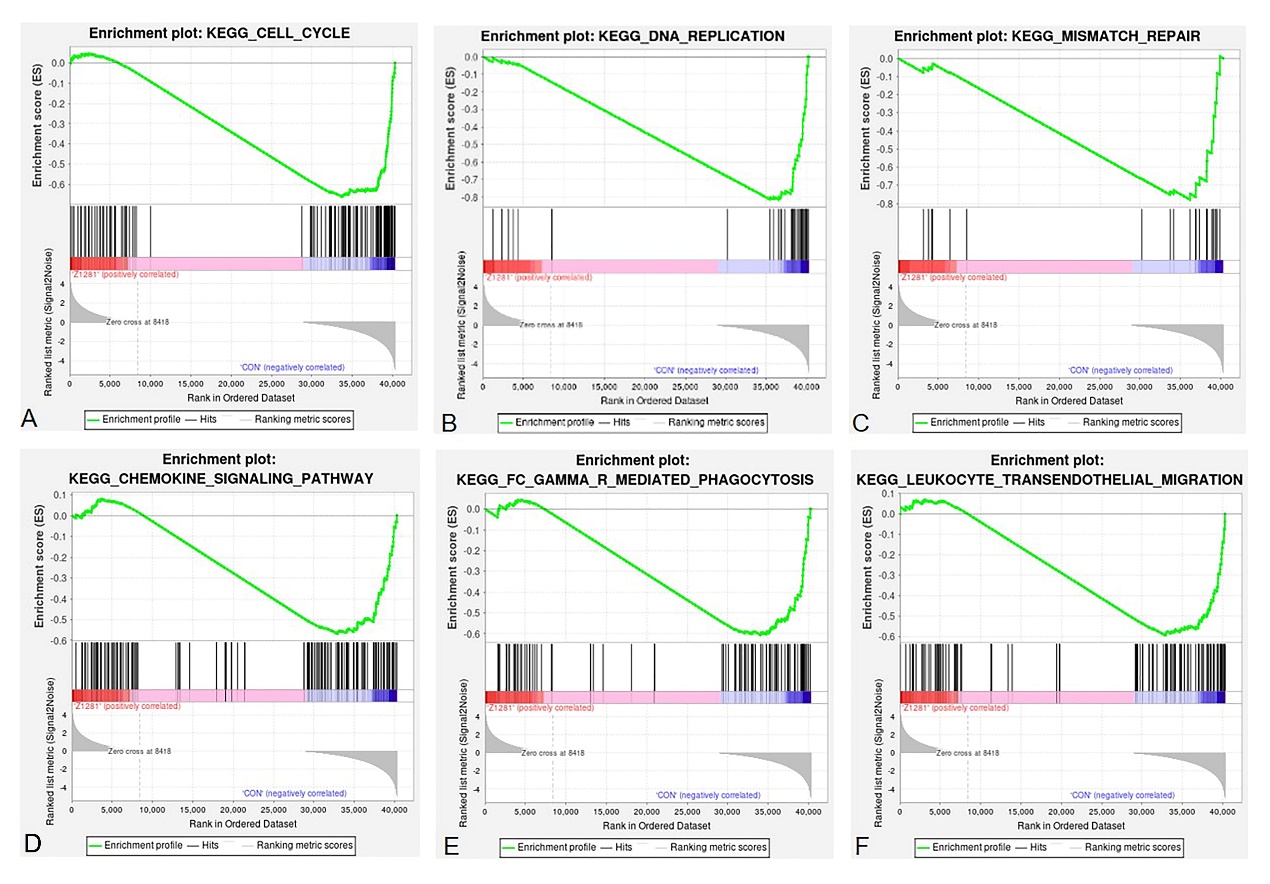


**Fig. S13.** Gene set enrichment analysis (GSEA) of **CHNQD-01281** treated T24 cell line.


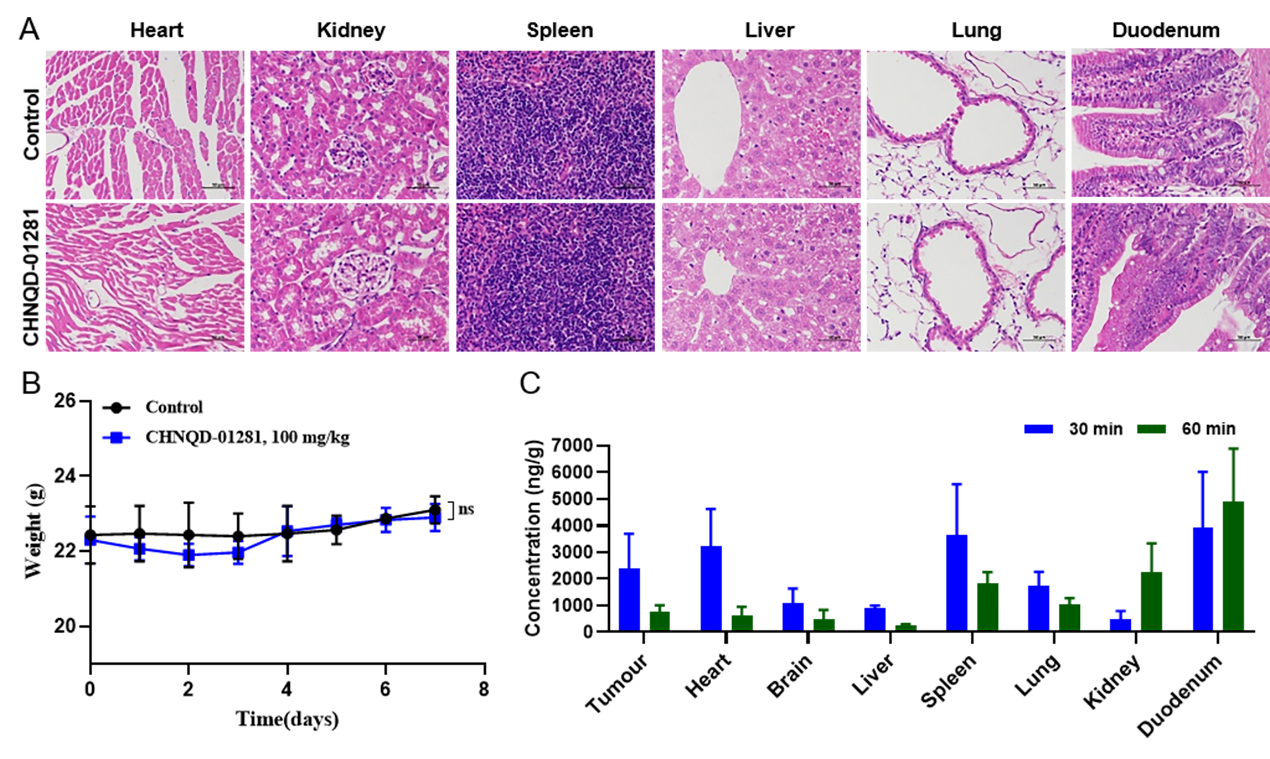


**Fig. S14.** Safety profile and tissue distribution of **CHNQD-01281** *in vivo*. **A** Representative H&E staining images (scale bar: 50 *μ*m) of main organs from vehicle and **CHNQD-01281** (100 mg/kg, ip.) treated C57BL/6 mice (*n* = 3). **B** Bodyweight evaluation of mouse after treatment with vehicle and **CHNQD-01281**. **C** Tissue distribution of **CHNQD-01281** in C57BL/6 mice (*n* = 3).


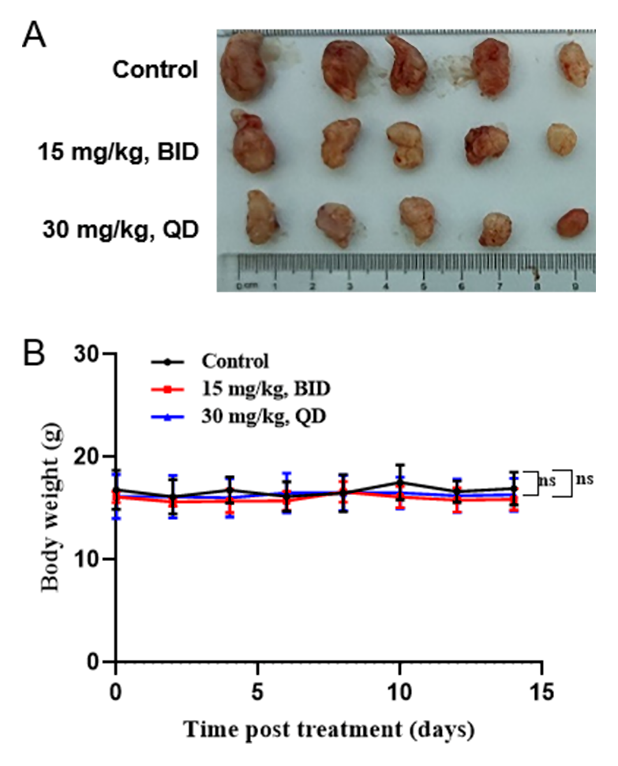


**Fig. S15.** **CHNQD-01281** inhibited T24 xenograft mouse model *in vivo*. **A** Visible tumor formation and photographs of representative tumors removed from mice at 14 days after initiation of treatment. **B** Mice bodyweight changes during treatment (*n* = 5).
